# Confinement-Induced Fractionation and Liquid–Liquid Phase Separation of Polymer Mixtures

**DOI:** 10.3390/polym15030511

**Published:** 2023-01-18

**Authors:** Arash Nikoubashman, Miho Yanagisawa

**Affiliations:** 1 Institute of Physics, Johannes Gutenberg University Mainz, Staudingerweg 7, 55128 Mainz, Germany; 2 Department of Mechanical Engineering, Keio University, Yokohama 223-8522, Japan; 3 Komaba Institute for Science, Graduate School of Arts and Sciences and Center for Complex Systems Biology, Universal Biology Institute, The University of Tokyo, Meguro, Tokyo 153-8902, Japan; 4 Graduate School of Science, The University of Tokyo, Bunkyo, Tokyo 113-0033, Japan

**Keywords:** phase separation, polymer mixture, droplet, protocell, confinement, molecular simulation, PEG, Dextran

## Abstract

The formation of (bio)molecular condensates via liquid–liquid phase separation in cells has received increasing attention, as these aggregates play important functional and regulatory roles within biological systems. However, the majority of studies focused on the behavior of pure systems in bulk solutions, thus neglecting confinement effects and the interplay between the numerous molecules present in cells. To better understand the physical mechanisms driving condensation in cellular environments, we perform molecular simulations of binary polymer mixtures in spherical droplets, considering both monodisperse and polydisperse molecular weight distributions for the longer polymer species. We find that confinement induces a spatial separation of the polymers by length, with the longer ones moving to the droplet center. This partitioning causes a distinct increase in the local polymer concentration near the droplet center, which is more pronounced in polydisperse systems. Consequently, the confined systems exhibit liquid–liquid phase separation at average polymer concentrations where bulk systems are still in the one-phase regime.

## 1. Introduction

The liquid–liquid phase separation (LLPS) of (bio)molecules in living cells has attracted much attention as a mechanism for intracellular organization via the formation of biomolecular condensates [1,2]. To elucidate the underlying mechanisms of LLPS, bulk solutions of purified biomolecules from cells have been analyzed extensively over the past decade. Although such in vitro studies facilitate the analysis of LLPS and comparisons with theoretical models [3], they systematically ignore confinement effects and the molecular diversity encountered in cellular environments [4]. For example, broad molecular weight distributions can have a profound impact on the phase behavior of polymers, e.g., leading to the self-assembly of monodisperse micelles from polydisperse surfactants [5] or the fractionation of polymer chains by molar mass in solutions near criticality [6,7]. Typically, the demixing of polymers becomes more pronounced in confinemenet due to the associated spatial inhomogeneity [8]. For example, simulations of binary polymer mixtures in spherical droplets have revealed an entropy-driven spatial segregation of the confined polymers based on their molecular weight, stiffness, and/or topology [9,10,11]. It is thus important to understand the phase behavior of confined systems for designing experiments and understanding the physical mechanisms driving LLPS in cellular environments.

Experimentally, confinement effects have been studied using DNA-based protocells [12,13] or water-in-oil droplets containing polymer mixtures such as polyethylene glycol (PEG) and bovine serum albumin, PEG and DNA, or PEG and Dextran, serving as a synthetic cytoplasm [14,15,16,17]. At sufficiently high concentrations, these polymer mixtures separate into two coexisting aqueous compartments, which can be used to localize additional components such as proteins [14,15]. Recently, Watanabe et al. systematically studied the phase coexistence of PEG–Dextran mixtures in cell-sized droplets, discovering that the two-phase coexistence region in small droplets extends to much lower PEG and Dextran concentrations compared to bulk systems [18]. The bulk behavior was only recovered for rather large droplets with radii R> 20 μm. They speculated that this *R*-dependent phase separation stemmed from a confinement-induced partitioning of the polymers. To elucidate the origin of this confinement-induced LLPS, we simulate PEG–Dextran mixtures in spherical confinement at two different droplet radii.

## 2. Simulation Model

**Figure 1:**
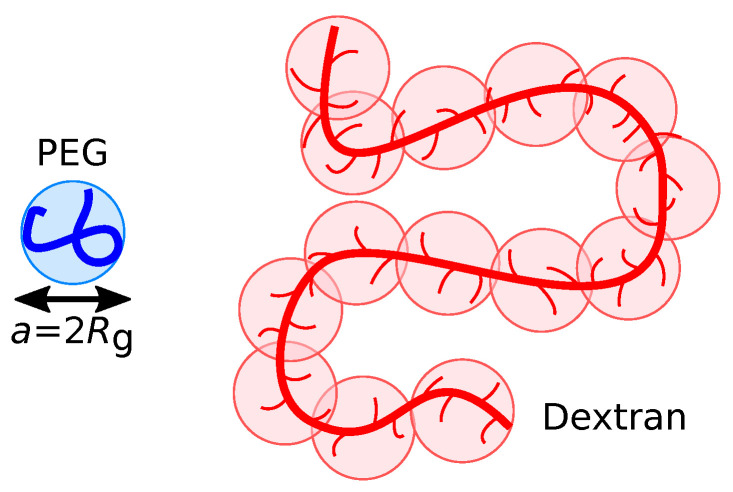
Schematic representation of the model, illustrating the coarse-graining of PEG and Dextran polymers.

We perform dissipative particle dynamics (DPD) simulations [19,20,21] using a coarse-grained polymer model in an explicit solvent. The simulations contain three different particle types, i.e., P (PEG), D (Dextran), and W (water), which are equal in their diameter *a* and mass *m*. To approach experimental length- and time-scales, we model each PEG chain as a single spherical bead of diameter *a*, as shown schematically in Figure 1. To facilitate a direct comparison with experiments, we have chosen polymer sizes similar to previously conducted experimental studies [18,22].

To establish a mapping between experiments and simulations, we first estimated the characteristic size of a PEG chain in solution: the mass and length of a Kuhn segment of PEG are $m_{K}^{P}=0.137\;\mathrm{kg}/\mathrm{mol}$ and $b_{K}^{P}=1.1\;\mathrm{nm}$, respectively, resulting in $N_{K}\equiv M_{w}/m_{K}\approx 44$ Kuhn segments for a PEG chain with molecular weight $M_{w}^{P}=6\;\mathrm{kg}/\mathrm{mol}$. At θ-conditions, the root-mean-square radius of gyration can be estimated as $R_{g}=b_{K}{\left(N_{K}/6\right)}^{1/2}$, resulting in a value of $R_{g}^{P}\approx 3.0\;\mathrm{nm}$. The DPD particles representing water have the same diameter *a*, and thus contain roughly 3700 water molecules. The Dextran chains are modeled as linear chains consisting of DPD particles with diameter *a* because the branches of Dextran are, on average, typically shorter than three glucose units [23] and therefore cannot be resolved at this level of coarse-graining (see Figure 1). We determined the number of DPD beads per Dextran chain, $N_{D}$, by matching $R_{g}^{D}$ from single-chain simulations to experimental $R_{g}$ measurements; in ref. [24], $R_{g}$ was derived from self-diffusion coefficient measurements of Dextran chains in water at T=293K, with molecular weights ranging between 4kg/mol and 464kg/mol. By extrapolating their data, we estimated $R_{g}=23.8\;\mathrm{nm}$ for a Dextran chain with 500kg/mol, which leads to $N_{D}=80$ for our simulation model. Hence, roughly 40 Dextran monomers are mapped to one DPD bead.

Thus, each DPD particle effectively represents a coil-like polymer segment, which typically interact with each other via soft and bounded pair potentials [25,26,27]. Therefore, we use the standard soft repulsion for the conservative forces acting between bonded and non-bonded DPD beads

(1) $$f_{m}\left(r\right)=\left\{\begin{array}{cc} A_{ij}\left(1-r/a\right)\hat{r}, & r\leq a\\ 0, & r>a, \end{array}\right.$$

where *r* is the distance between the two particles, and $\hat{r}$ is the unit vector connecting the two. The parameter $A_{ij}$ controls the repulsion strength between particles of type *i* and *j* and has been set according to [21]

(2) $$A_{ij}=A_{ii}+3.497\chi_{ij},$$

with $A_{ii}=25\;k_{B}T/a$ and Flory–Huggins interaction parameter $\chi_{ij}$ (the values for $\chi_{ij}$ are discussed in Section 3.1 below). This specific value for $A_{ii}$ was originally determined by Groot and Warren to match the compressibility of water [21], but since then, this choice has been widely used to model other (incompressible) liquids and polymer melts [28,29,30,31].

Neighboring monomers within a Dextran chain are bonded through harmonic springs with force

(3) $$f_{b}\left(r\right)=-kr.$$

Note that the individual beads should not be regarded as single monomers, but rather as fluid elements containing several chain segments. Thus, the harmonic springs between DPD particles do not represent covalent bonds between monomers, but instead ensure the connectivity of the Dextran chains. We use a soft spring constant $k=4\;k_{B}T/a^{2}$ [28], which is consistent with the typical free energy associated with deforming a polymer in the blob model [32].

In addition to these two conservative forces, all particles are subjected to pairwise dissipative and random forces

(4) $$f_{d}\left(r\right)=-\gamma_{ij}\omega \left(r\right)\left(\hat{r}\cdot \Delta v\right)\hat{r},$$

(5) $$f_{r}\left(r\right)=\sqrt{\gamma_{ij}\omega \left(r\right)}\xi \hat{r},$$

with drag coefficient $\gamma_{ij}$, and velocity difference between two particles Δv. The parameter ξ is a uniformly distributed random number drawn for each particle pair, with zero mean $\langle \xi (t)\rangle =0$ and variance $\langle \xi \left(t\right)\xi \left(t^{\prime }\right)\rangle =2k_{B}T\delta \left(t-t^{\prime }\right)$ to satisfy the fluctuation-dissipation theorem. For simplicity, we used the same drag coefficient $\gamma_{ij}=\gamma =4.5\;\sqrt{mk_{B}T}/a$ for all particles, and the standard DPD weight function [21]

(6) $$\omega \left(r\right)=\left\{\begin{array}{cc} {\left(1-r/a\right)}^{2}, & r\leq a\\ 0, & r>a. \end{array}\right.$$

For the droplet simulations, all beads are confined to a spherical container with radius *R* by applying a purely repulsive Weeks–Chandler–Andersen (WCA) potential [33]

(7) $$\begin{array}{c} U_{\mathrm{WCA}}\left(r^{\prime }\right)=\left\{\begin{array}{cc} 4k_{B}T\left[{\left(\frac{a}{r^{\prime }}\right)}^{12}-{\left(\frac{a}{r^{\prime }}\right)}^{6}+\frac{1}{4}\right], & r^{\prime }\leq 2^{1/6}a\\ 0, & r^{\prime }>2^{1/6}a \end{array}\right., \end{array}$$

where $r^{\prime }$ is the distance between the droplet surface and the center of a bead. In all simulations, the particle number density was set to $\rho =3\;a^{-3}$. The equations of motion were integrated using a time step of Δt=0.02τ, with the unit of time being τ. Each simulation was run for at least $10^{7}$ time steps, and three independent simulations were performed for each parameter set to improve the statistics and determine measurement uncertainties.

## 3. Results

### 3.1. Parameterization and Bulk Phase Behavior

To faithfully reproduce experimental conditions, we first needed to determine the interaction parameters for the PEG (P), Dextran (D), and water (W) particles (see Section 2). Following previous simulation studies [34,35], we used a Flory–Huggins interaction parameter of $\chi_{P-W}=0.3$ for the PEG-water interactions, which was extracted from experimental phase coexistence measurements by Saeki et al. [36]. We took $\chi_{D-W}=0.50$ for the Dextran–water interactions, derived from experimental vapor-pressure measurements at T=298K conducted by Bercea et al. [37]. This value is in excellent agreement with previous findings by Clark [38], who applied a Flory–Huggins theory-based analysis to experimental tie line data of PEG–Dextran mixtures in water at T=298−300K [39,40]; Clark also extracted the PEG–Dextran interaction parameter from his analysis, i.e., $\chi_{P-D}=0.031\pm 0.007$. Note that the physically relevant quantity for a pair of polymers is the *combined* Flory–Huggins parameter χN [32], and thus a scaled interaction parameter $\chi_{P-D}^{\mathrm{eff}}\approx 1.23$ needs to be used in the simulations to reach the same ${\left(\chi N\right)}_{P-D}\approx 100$ as in the experiments, since we mapped about 40 Dextran monomers onto one DPD particle. Given the uncertainties in extracting $\chi_{ij}$ from experiments, and the high degree of coarse-graining of our model, it was, however, unclear whether this initial parameterization would faithfully reproduce the interactions between the PEG and Dextran chains in water.

**Figure 2:**
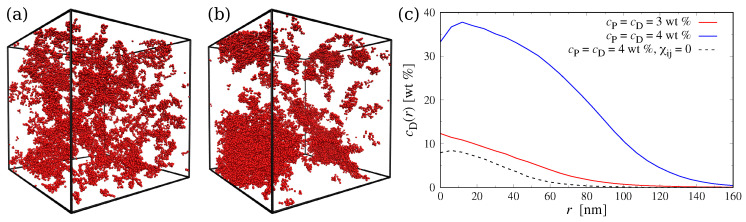
(**a**,**b**) Simulation snapshot of the bulk system at (**a**) $c_{P}=3\;\mathrm{wt}\%$, $c_{D}=3\;\mathrm{wt}\%$, and (**b**) $c_{P}=4\;\mathrm{wt}\%$, $c_{D}=4\;\mathrm{wt}\%$. Only Dextran beads shown for clarity. Snapshots rendered using Visual Molecular Dynamics (version 1.9.3) [42]. (**c**) Radial concentration profile of Dextran monomers $c_{D}\left(r\right)$ in the largest Dextran aggregate.

To test (and tune, if necessary) the interaction parameters of our coarse-grained model, we first attempted to reproduce the experimentally known phase behavior [18,41] of aqueous PEG–Dextran mixtures in the bulk. The groups of Dimova [41] and Yanagisawa [18] determined the binodals of mixtures containing short PEG chains ($M_{w}^{P}=6$ or 8kg/mol) and long Dextran chains ($M_{w}^{D}\approx 500\;\mathrm{kg}/\mathrm{mol}$), finding a critical concentration of slightly below 4wt% for PEG and 4wt% for Dextran. Initially, we simulated bulk systems at two concentrations, i.e., $c_{P}=3\;\mathrm{wt}\%$ for PEG and $c_{D}=3\;\mathrm{wt}\%$ for Dextran, where we expected a single mixed phase, and at $c_{P}=4\;\mathrm{wt}\%$ and $c_{D}=4\;\mathrm{wt}\%$, where the polymers should phase separate. We performed simulations in a cubic box with an edge length of L≈360nm and applied periodic boundary conditions to all three Cartesian directions. The systems were initialized by placing all PEG particles and Dextran chains in opposite halves of the simulation box and were then run until the density profiles did not change anymore. For $\chi_{P-D}^{\mathrm{eff}}=1.23$, we found a single phase at both concentrations, which indicates that the initially chosen $\chi_{P-D}^{\mathrm{eff}}$ value was too small. Therefore, we iteratively increased $\chi_{P-D}^{\mathrm{eff}}$ until we observed aggregation of the Dextran chains at the higher concentration and observed a fully mixed system at the lower concentration. This was achieved for $\chi_{P-D}^{\mathrm{eff}}=18.6$ (Figure 2), which is about 15 times larger than our initial estimate for the PEG–Dextran interactions.

To quantify the size and concentration of the Dextran condensates, we performed a cluster analysis using the density-based spatial clustering of applications with noise (DBSCAN) algorithm [43]; here, Dextran monomers are assigned to the same aggregate if their distance is smaller than 7nm, which roughly corresponds to the position of the first minimum of the radial distribution function g(r) between Dextran particles and all other particle types. To establish a baseline, we performed additional simulations of ideal mixtures by setting $\chi_{ij}=0$. Figure 2c shows the radial concentration profile $c_{D}\left(r\right)$ of Dextran monomers belonging to the largest cluster identified in the system. For the system at $c_{P}=4\;\mathrm{wt}\%$ and $c_{D}=4\;\mathrm{wt}\%$, this analysis revealed one large droplet with a Dextran concentration of about 35wt% in its core. In contrast, the cluster identified at $c_{P}=3\;\mathrm{wt}\%$ and $c_{D}=3\;\mathrm{wt}\%$ was much smaller and more diluted, and the resulting concentration profile resembled that of an ideal Dextran chain in solution. To characterize the size of the aggregates in more detail, we also computed the average number of Dextran monomers in a cluster, $\langle M\rangle$. For the the ideal mixtures, this analysis yielded ${\langle M\rangle }_{0}\approx 100$, which is comparable to the number of monomers per Dextran chain ($N_{D}=80$). For the less concentrated system with $\chi_{ij}\neq 0$, we found $\langle M\rangle \approx 400$, which indicates the formation of small intermittent clusters. In contrast, at $c_{P}=4\;\mathrm{wt}\%$ and $c_{D}=4\;\mathrm{wt}\%$, we found a much larger value of $\langle M\rangle \approx 4800$.

**Figure 3:**
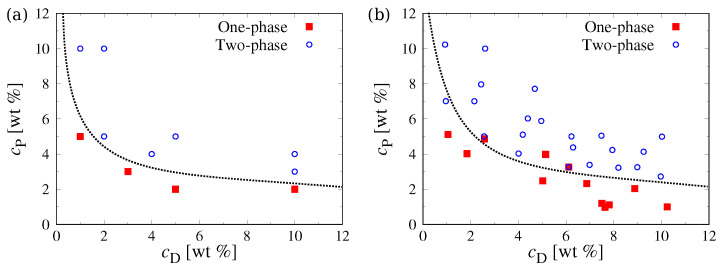
Phase diagram of PEG–Dextran blends in bulk from (**a**) simulations and (**b**) experiments [18]. The dotted lines indicate the estimated binodal. Simulation data are generated from monodisperse systems with $M_{n}^{P}=6\;\mathrm{kg}/\mathrm{mol}$ and $M_{n}^{D}=500\;\mathrm{kg}/\mathrm{mol}$, while experimental data were gathered for blends with $M_{w}^{P}=6\;\mathrm{kg}/\mathrm{mol}$ and $M_{w}^{D}=500\;\mathrm{kg}/\mathrm{mol}$.

The discrepancy between the initial estimate and the final value of $\chi_{P-D}^{\mathrm{eff}}$ is rather large, and we can only speculate about its origin: Clark extracted $\chi_{P-D}$ from experimental coexistence curves using Flory–Huggins solution theory [38], which ignores the polymer architecture and thus the branching of the Dextran chains. Further, a monodisperse molecular weight distribution is assumed in his treatment, although generally available Dextran polymers typically have a broad molecular weight distribution [7,17,18,22,44]. Finally, we used a rather coarse-grained description, which maps about 40 monomers onto a single bead, resulting in a larger entropy of mixing in the simulations compared to the experiments [32]. To compensate for these effects, we will use $\chi_{P-D}^{\mathrm{eff}}=18.6$ in the following. Figure 3 shows the resulting phase coexistence results from our simulations compared to experiments [18], which are in excellent agreement with each other, thus further corroborating the appropriate parameterization of our simulation model.

### 3.2. Phase Behavior in Confinement

In recent experiments by Watanabe et al., the confinement-induced phase separation of PEG–Dextran mixtures was observed for droplets with radii *R* < 20 μm [18]. Simulating such large droplets is computationally infeasible, even at the employed level of coarse-graining (see Methods section), as roughly $7\times 10^{9}$ particles would already be needed to represent a droplet with R=5μm. Therefore, we performed simulations at two smaller radii, i.e., R≈260nm and R≈380nm, which should still allow us to capture the effect of confinement on the phase behavior. Further, we considered mixtures containing either monodisperse or polydisperse Dextran chains, since high-molecular-weight Dextran usually has a broad molecular weight distribution; for example, the Dimova group used Dextran chains with $M_{w}\approx 380-490\;\mathrm{kg}/\mathrm{mol}$ and dispersities in the range of $Đ\equiv M_{w}/M_{n}\approx 1.8-2.2$ [7,41,44], while the Yanagisawa group used Dextran with $M_{w}\approx 500\;\mathrm{kg}/\mathrm{mol}$ and Đ≈3.1 [17,18]. In our simulations with polydisperse Dextran chains, we drew the molecular weight of each polymer from a Gaussian distribution, targeting Đ≈1.5 and $M_{n}\approx 500\;\mathrm{kg}/\mathrm{mol}$. The PEG chains were kept monodisperse throughout, which is consistent with the rather small polydispersity of Đ≈1.1 reported in the experimental literature for low-molecular-weight PEG [7,17,44]. In all simulations, we selected the number of PEG and Dextran chains so that $c_{P}=3\;\mathrm{wt}\%$ and $c_{D}=3\;\mathrm{wt}\%$, averaged over the entire droplet volume, which lies in the mixed one-phase regime of the bulk phase diagram (see Section 3.1).

**Figure 4:**
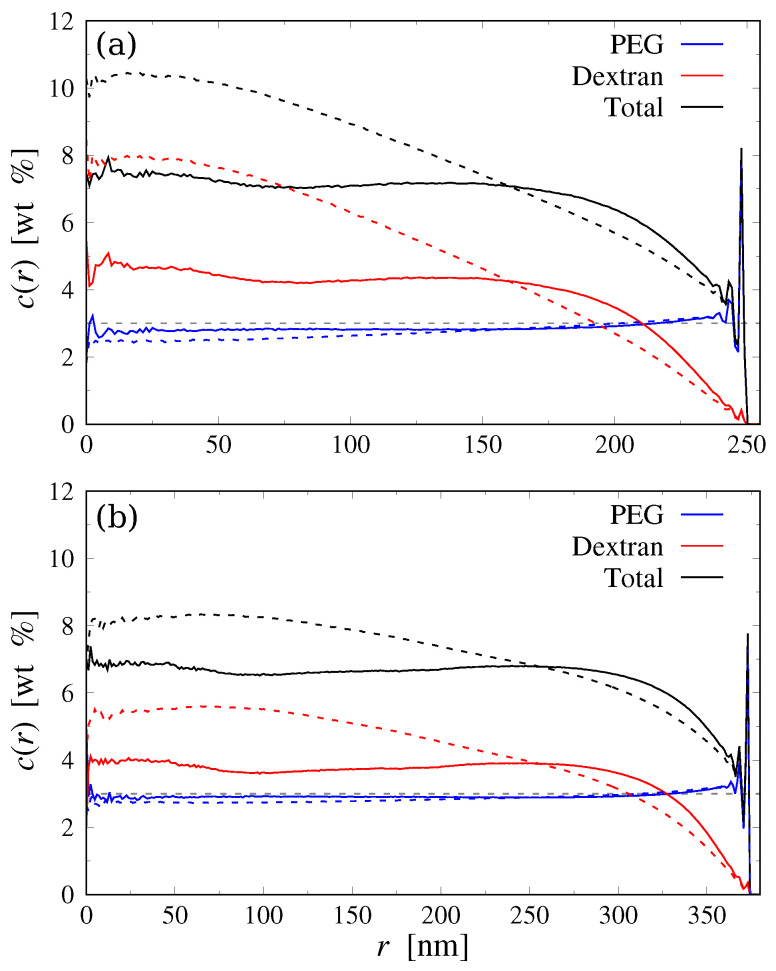
Radial monomer concentration profiles for droplets with (**a**) R≈260nm and (**b**) R≈380nm. Solid and dashed lines show simulation results for monodisperse and polydisperse Dextran chains, respectively. The horizontal dashed grey line indicates the average polymer concentration of each species (3wt%).

To study the spatial distribution of the PEG and Dextran polymers in the droplet, we first calculated the radial monomer concentration profiles c(r) of the two species (Figure 4), which reveal several important features: (i) in all cases, there is a local surplus and layering of PEG near the droplet surface, which is typical for short molecules close to hard walls [45]. In contrast, the long Dextran polymers are depleted from the droplet surface because of the associated loss in conformational entropy in that region [11]. In the monodisperse case, the width of this depletion zone is roughly $2\;R_{g}^{D}\approx 50\;\mathrm{nm}$, with $R_{g}^{D}\approx 23.8\;\mathrm{nm}$ being the radius of gyration of a Dextran chain at infinite dilution. In contrast, the excluded region is much wider for the polydisperse case due to the broader $R_{g}^{D}$ spectrum; (ii) as a result, the Dextran concentration in the droplet center becomes distinctly larger than the average value (3wt%), reaching almost 8wt% for the polydisperse case in the small droplets (see Figure 4). By comparison, the concentration of PEG chains near the droplet center is only slightly below the average. (iii) As expected, the effect of confinement is significantly more pronounced in the smaller droplet, since the region close to the droplet surface occupies a larger volume fraction, i.e., $1-{\left(R-2R_{g}^{D}\right)}^{3}/R^{3}\approx 0.45$ for R=260nm vs. ≈0.33 for R=380nm.

**Figure 5:**
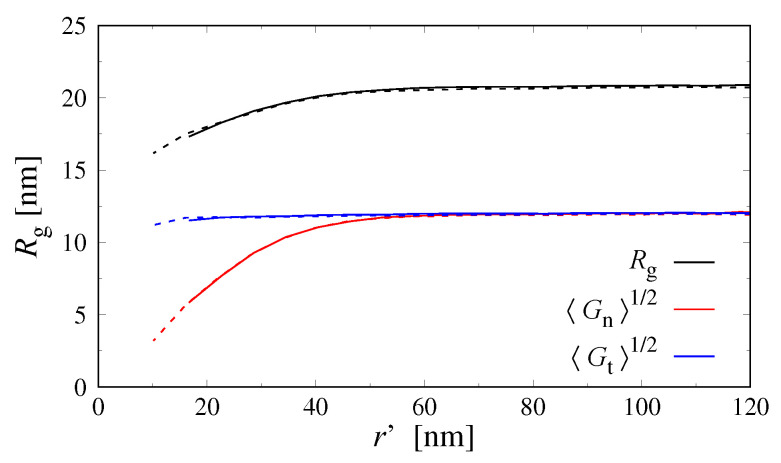
Radial profiles of the radius of gyration of monodisperse Dextran, $R_{g}$, and its normal (${\langle G_{n}\rangle }^{1/2}$) and tangential (${\langle G_{t}\rangle }^{1/2}$) components relative to the droplet surface. Data plotted against the distance between the droplet surface and the polymer’s center of mass, $r^{\prime }$. Solid and dashed lines show results for R≈260nm and R≈380nm, respectively.

We analyzed the shape of the confined Dextran chains by computing their radius of gyration tensor

(8) $$G=\frac{1}{N_{D}}\sum_{i}^{N_{D}}\Delta r_{i}\Delta r_{i}^{T},$$

with $\Delta r_{i}$ being the position of monomer *i* relative to the polymer’s center of mass. The root-mean-square radius of gyration is then $R_{g}\equiv {\langle R_{g}^{2}\rangle }^{1/2}={\langle G_{n}+2G_{t}\rangle }^{1/2}$, where $G_{n}$ and $G_{t}$ are the components of G normal and tangential relative to the droplet surface, respectively. Figure 5 shows these components for monodisperse Dextran vs. the distance between the polymer’s center of mass and the droplet surface. Polymers at distances larger than $\approx 2\;R_{g}$ have isotropic shapes, ${\langle G_{n}\rangle }^{1/2}={\langle G_{t}\rangle }^{1/2}\approx 12\;\mathrm{nm}$, whereas they become increasingly compressed along the normal direction as they approach the droplet surface (the tangential component is nearly constant). Further, chain conformations in the small and large droplets are almost indistinguishable.

**Figure 6:**
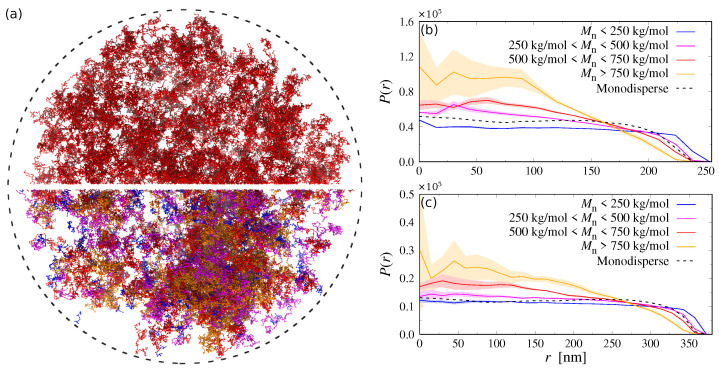
(**a**) Simulation snapshot of the confined systems (R≈380nm, indicated by dashed circle). The top half shows the monodisperse case with Dextran chains colored in red. The bottom half shows the polydisperse case with Dextran chains colored according to their molecular weight as in panels (**b**,**c**). Water and PEG particles have been omitted for clarity. (**b**,**c**) Probability P(r) to find a Dextran chain in the specified molecular weight range at center-of-mass position *r* in a droplet with (**b**) R≈260nm and (**c**) R≈380nm. The shaded area indicates the standard deviation from three independent runs. The dashed black line shows the distribution of the monodisperse case.

The confinement-induced increase in the polymer concentration near the droplet center could induce a phase separation of the PEG and Dextran chains there, as the local polymer concentration might cross the binodal of the mixtures (cf. Figure 3). Figure 6 shows simulation snapshots for the monodisperse and polydisperse systems confined in large droplets: while the monodisperse system appears to be still in the mixed one-phase regime, we can clearly see large Dextran aggregates in the polydisperse case, which is in excellent agreement with recent experiments by Watanabe et al. [18]. Looking more closely at the simulation snapshot, we can see that the aggregates in the polydisperse systems primarily consist of longer Dextran chains, whereas the shorter ones still remain well dispersed. Zhao et al. observed a similar molar mass fractionation in aqueous two-phase polymer solutions of PEG and Dextran [7], where the longer Dextran chains accumulated in the Dextran-rich phase, while the shorter Dextran chains were contained in the PEG-rich phase.

To better understand the distinct differences between the behavior of the monodisperse and polydisperse mixtures, we determined the probability P(r) of finding Dextran chains at center-of-mass position *r*, itemized by their molecular weight. These results are shown in Figure 6b,c for both droplet sizes, revealing a distinct spatial fractionation of the Dextran chains: short polymers with $M_{n}<250\;\mathrm{kg}/\mathrm{mol}$ are distributed almost homogeneously throughout the droplet and also come much closer to the droplet surface compared to the longer chains. In contrast, longer Dextran chains are moving to the droplet center to maximize their conformational entropy. These findings are consistent with recent experiments [18], where Watanabe et al. inferred from surface tension measurements that short Dextran chains ($M_{w}\ll 500\;\mathrm{kg}/\mathrm{mol}$) preferentially adsorbed to the droplet surface. This radial partitioning of short and long Dextran chains promotes their phase separation, as longer chains have a distinctly smaller entropy of mixing compared to their shorter counterparts [32].

**Figure 7:**
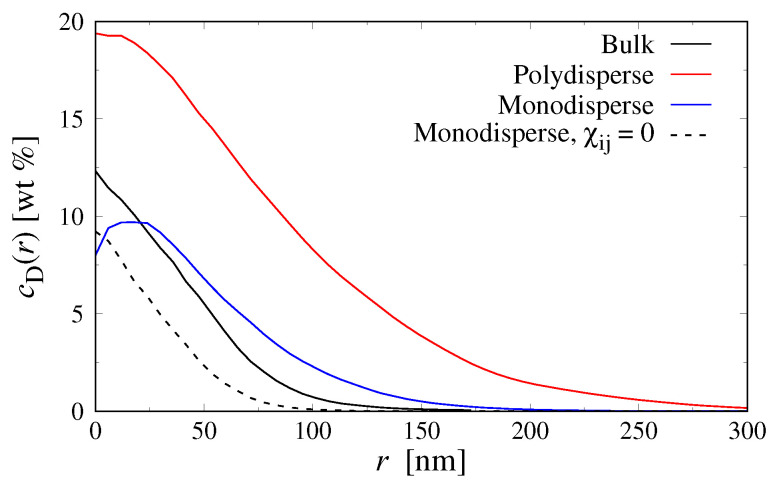
Radial concentration profile of Dextran monomers $c_{D}\left(r\right)$ in the largest Dextran aggregate in bulk and droplet systems (R≈380nm) at overall average concentration $c_{P}=3\;\mathrm{wt}\%$ and $c_{D}=3\;\mathrm{wt}\%$.

Finally, we quantified the size of the aggregates through the radial concentration profile $c_{D}\left(r\right)$ of Dextran monomers belonging to the largest cluster, and through the average number of Dextran monomers in a cluster, $\langle M\rangle$, as explained in Section 3.1 above. To establish a reference, we performed additional simulations of ideal mixtures by setting $\chi_{ij}=0$ and determined the corresponding mean aggregation number ${\langle M\rangle }_{0}$. The concentration profiles are shown in Figure 7, revealing that the Dextran clusters in the polydisperse confined systems are much larger and more concentrated compared to the monodisperse systems in bulk as well as in confinement.

Table 1 summarizes the results of $\langle M\rangle$ for all droplet simulations, which will be discussed in the following: For ideal mixtures with monodisperse Dextran chains, we find ${\langle M\rangle }_{0}\approx 100$, which is comparable to the number of monomers per Dextran chain ($N_{D}=80$). This value is sensible given that $\chi_{ij}=0$ and that the search radius of the clustering algorithm is slightly larger than the average segment length of our Dextran model (b≈5.5nm). For polydisperse Dextran in the smaller droplets (R=260nm), the mean aggregation number increases to ${\langle M\rangle }_{0}\approx 380$ due to an accumulation of longer Dextran chains in the droplet center, even at ideal conditions. This effect is considerably less pronounced in the larger droplets (cf. Figure 6), where we find ${\langle M\rangle }_{0}\approx 110$ instead. For the non-ideal mixtures with monodisperse Dextran, we find $\langle M\rangle \approx 1300-1600$, which indicates the existence of small aggregates consisting of 15−20 Dextran chains (there are, in total, 348 and 1175 Dextran chains in the small and large droplets, respectively). The mean aggregation number becomes significantly larger in polydisperse PEG–Dextran mixtures, reaching values up to $\langle M\rangle \approx$ 10,000. Interestingly, $\langle M\rangle$ is about two times smaller in the smaller droplets (see Table 1), which is likely a finite-size effect, as the smaller droplets contain about three times fewer Dextran chains compared to the large droplets. Nevertheless, both systems show clearly that the confinement-induced fractionation of short and long Dextran chains drives phase separation, as observed in recent experiments [18].

## 4. Conclusions

To better understand the phase behavior and conformations of (bio)polymers in droplets, we have performed coarse-grained molecular simulations of binary polymer mixtures in spherical confinement. We have parameterized our model to closely mimic the behavior of aqueous PEG–Dextran mixtures and have considered both monodisperse and polydisperse molecular weight distributions for the Dextran chains. Simulations have been conducted for two droplet sizes at polymer concentrations lying in the mixed one-phase region in bulk. In spherical confinement, we have found a distinct spatial separation of the polymers by length, with the longer ones accumulating at the droplet center to maximize their conformational entropy. Furthermore, chains near the droplet surface became increasingly compressed along their normal direction. This confinement-induced partitioning was much more pronounced in the polydisperse systems and caused the phase separation of the two polymer species at average polymer concentrations where the bulk system was still in the one-phase regime.

Although we have chosen the model parameters to replicate PEG–Dextran mixtures, the rather generic nature of our coarse-grained model makes our results applicable to a wide range of different polymer mixtures. Our simulations demonstrate how the distribution of polymers is affected by confinement effects, even at good solvent conditions, with longer chains moving to the droplet center to maximize entropy. The resulting spatial inhomogeneity can drastically alter the phase behavior of the confined polymers, which is important for understanding, e.g., the liquid–liquid phase separation of biopolymers in cellular environments. Furthermore, our simulations provide useful guidelines for the fabrication of polymer-loaded droplets. For example, by tuning the interactions between the droplet surface and the different polymer species, one can either enhance or suppress their spatial separation and thus control the resulting phase behavior and surface tension.

## Figures and Tables

**Table 1:** Mean number $\langle M\rangle$ of Dextran segments in a Dextran aggregate (${\langle M\rangle }_{0}$ corresponds to ideal mixtures with $\chi_{ij}=0$).

	Monodisperse		Polydisperse	
R	$\langle M\rangle$	${\langle M\rangle }_{0}$	$\langle M\rangle$	${\langle M\rangle }_{0}$
260nm	1600±60	100±5	5300±1200	380±20
380nm	1300±30	97±4	9800±1600	110±3

## Data Availability

The data that support the findings of this study are available from the corresponding author upon reasonable request.
